# Cytokine-induced killer cells: A novel treatment for allergic airway inflammation

**DOI:** 10.1371/journal.pone.0186971

**Published:** 2017-10-26

**Authors:** Panwadee Pluangnooch, Sunita Timalsena, Adisak Wongkajornsilp, Kitipong Soontrapa

**Affiliations:** Department of Pharmacology, Faculty of Medicine Siriraj Hospital, Mahidol University, Bangkok, Thailand; Forschungszentrum Borstel Leibniz-Zentrum fur Medizin und Biowissenschaften, GERMANY

## Abstract

The effectiveness of cytokine-induced killer (CIK) cells for treatment of cancers has long been appreciated. Here, we report for the first time that CIK cells can be applied to treat allergic airway inflammation. Adopting from an established protocol with some modifications, we generated CIK cells ex vivo from mouse T cells, and examined their effectiveness in treatment of allergic airway inflammation using the ovalbumin-induced model of allergic airway inflammation. Based upon evaluation of bronchoalveolar lavage cellularity, T helper type2 cytokine levels and lung histology, all of which are important parameters for determining the severity of allergic airway inflammation, diseased mice treated with CIK cells showed significant reductions in all the parameters without any obvious adverse effects. Interestingly, the observed effects were comparable to those treated with dexamethasone. Thus, our study provides a novel application of CIK cells in treatment of allergic airway inflammation.

## Introduction

Cytokine-induced killer (CIK) cells, a widely studied cell-based immunotherapy for cancer treatment, are generated ex vivo by culturing peripheral blood mononuclear cells (PBMC) with the timely addition of interferon gamma (IFN- γ), monoclonal antibody against CD3 (anti-CD3) and interleukin-2 (IL-2) for 3–4 weeks. CIK cells are heterogeneous cells with the major effector population identified as CD1d independent CD8^+^ NKT cells expressing CD3^+^ CD8^+^ CD56^+^ (for murine CIK cells: CD3^+^ CD8^+^NK1.1^+^). Their anti-tumor activity has been reported to be due to high productions of Th1 cytokine (IFN-γ) and cytolytic granules (perforin and granzymes) in a non-major histocompatibility complex (MHC)-restricted manner [1–4]. Because of these Th1 and NK dual characteristics, CIK cell-based immunotherapy showed promising results in treatment of several types of hematologic and solid cancers in humans, and also proved to be highly safe for clinical use as evidenced by many clinical studies [5–10].

Allergic asthma, one of the major public health concerns nowadays, is a chronic disease of allergic airway inflammation. In general, bronchodilators and corticosteroids are used to relieve symptoms of allergic asthma [11]. However, these drugs, especially corticosteroids, cause several adverse effects, such as osteoporosis, metabolic imbalance, glaucoma, and immunosuppression. Accordingly, it is very beneficial to find a new therapeutic approach for millions of asthmatic patients. As the pathophysiology of allergic airway inflammation is driven mainly by T helper type2 (Th2) immune responses, one of novel strategies is to encourage Th1 responses with cell-based immunotherapy in order that it can suppress Th2 immune responses [12,13]. Previous preclinical studies with adoptive transfer of Th1 cells showed that this strategy could effectively alleviate pathologies of allergic airway inflammation in mice. A Th1 cytokine, IFN-γ, was reported to be a major factor in this Th2-counter regulation [14–16]. Nevertheless, other studies applying this approach showed opposite results [17,18]. Given these conflicting results, application of Th1 cells to treat allergic airway inflammation is still a matter of debates. Recently, natural killer (NK) cells are reported to play an important role in resolution of allergic airway inflammation by clearing eosinophil and antigen-specific T cells [19]. That CIK cells are endowed with both Th1 and NK cell properties indicates that they could be a potential candidate for cell-based immunotherapy of allergic airway inflammation. In this study, we examined the effectiveness of CIK cells in treatment of allergic airway inflammation using the mouse model of ovalbumin-induced allergic airway inflammation.

## Materials and methods

### Materials

Recombinant mouse IFN-γ, anti-mouse CD3e, recombinant mouse IL-2, FITC conjugated anti-mouse CD4, PE conjugated anti-mouse IFN-γ, granzyme B, perforin, PE-Cy5 conjugated anti-mouse CD8a, APC conjugated anti-mouse NK1.1 and isotype controls of each antibody were purchased from eBioscience. FITC conjugated anti-mouse CD3e, PE conjugated anti-mouse IL-4 and IL-5 and isotype controls of each antibody were purchased from BioLegend. R-PE labeled CD1d tetramer preloaded with α-GalCer and R-PE labeled CD1d tetramer negative control were purchased from Proimmune. The mouse IFN-γ, IL-4, IL-5 and IL-13 ELISA kits were purchased from eBioscience. Alum adjuvant was purchased from Thermo Fisher Scientific. Ovalbumin was purchased from Sigma-Aldrich. The endotoxin level in ovalbumin is < 1 EU/mg.

### Animals

5–6 week-old C57BL/6 and BALB/c male mice were obtained from National Laboratory Animal Center (Mahidol University, Bangkok, Thailand). The mice were sacrificed by intraperitoneal injection of pentobarbital (50 mg/kg). All experiments were performed in accordance with the guidelines of Mahidol University and the Office of the National Research Council of Thailand (NRCT) and approved by the Committee on Animal Care and Use of Siriraj hospital (SiACUC).

### Generation of CIK cells

CIK cells were generated from thymocytes as described previously [20]. Briefly, thymus glands were removed from naive mice and dispersed through a cell strainer to prepare single-cell suspension. Thymocytes at 3x10^6^ cells per ml were suspended in RPMI 1640 medium supplemented with 10% fetal bovine serum (FBS), 1000 U/mL recombinant mouse IFN-γ, 100 U/mL penicillin and 100 μg/mL streptomycin at 37°C in a 5% CO_2_ incubator for 24 hours. Next, cells were transferred to a culture dish coated with 100 ng/mL of anti-CD3 MAb with addition of 300 IU/mL recombinant mouse IL-2. Complete RPMI supplemented with 50 IU/mL recombinant mouse IL-2 was added every 3 days. On day 21, cells were collected.

For evaluation of cell characteristics, cell surface proteins, intracellular cytokines and cytotoxic granules were checked with flow cytometry. To measure secreted cytokines, 2 x 10^6^ cells of CIK cells or thymocytes were suspended in 1 mL of RPMI 1640 medium supplemented with 10% fetal bovine serum (FBS), 100 U/mL penicillin and 100 μg/mL streptomycin in a 24-well plate at 37°C in a 5% CO_2_ incubator for 48 hours. This assay was repeated on four separate occasions in duplicate. The supernatants were collected. Cytokines in the supernatants were measured by ELISA according to the procedures recommended by the manufacturer.

### Generation of the mouse model of allergic airway inflammation

**Fig 1:**
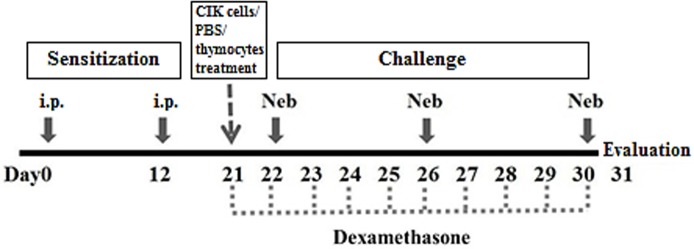
A diagram of the protocol for the mouse model of allergic airway inflammation and CIK cells treatment. C57BL/6 mice were sensitized with an intraperitoneal (i.p.) injection of 50 μg ovalbumin and 25 μg Alum on days 0 and 12. On days 22, 26 and 30, mice were challenged with 2% OVA aerosol (w/v) in saline (Neb) for 30 min. For treatments, 1x10^7^ of CIK cells, PBS or thymocytes were transferred via tail vein on day 21, and 2mg/kg/day dexamethasone was injected i.p. from day 21 to 30. All mice were evaluated 24 h after last challenge.

Mice were sensitized with an intraperitoneal (i.p.) injection of 0.3 mL phosphate buffer saline (PBS) containing 50 **μ**g ovalbumin (OVA) and 25 μg alum on days 0 and 12. On days 22, 26 and 30, allergic groups were challenged with nebulized 2%OVA (w/v) in saline for 30 minutes [21]. Normal control mice were both sensitized and challenged with PBS. 1x10^7^ CIK cells were transferred via tail vein on day 21. For therapeutic control, 2 mg/kg/day dexamethasone was given i.p. every day from day 21 to 30. Mice were evaluated 24 hours after last challenge (Fig 1).

### Evaluation of serum and bronchoalveolar lavage fluid

Allergic airway inflammation was induced in mice as mentioned above. Twenty four hours after the final exposure to OVA nebulization, mice were anesthetized with pentobarbital (50 mg/kg). Serum was collected via retro-orbital bleeding. Lungs were lavaged with 1 mL PBS for three times and cells in the bronchoalveolar lavage (BAL) were counted using hemocytometer. The differential cell counts were prepared using Cytospin (Thermo Fisher Scientific, USA). BAL cells stained with methylene blue and eosin were determined based on light microscopic evaluation (>300 cells/slide). The concentrations of IL-5, IL-13 in the serum and BAL were measured by ELISA.

### Lung histological analysis

Following bronchoalveolar lavage, the lungs were removed and fixed with 4% paraformaldehyde, embedded in paraffin, sectioned and stained with haematoxylin and eosin (H&E) or periodic acid-Schiff (PAS). Semiquantitative scoring systems were used to grade the extent of lung inflammation and goblet cell hyperplasia. Briefly, to determine the severity of inflammatory cell infiltration, cell counts were performed blindly based on a five-point scoring system: 0, no cell; 1, a few cells; 2, a ring of inflammatory cells 1 cell layer deep; 3, a ring of inflammatory cells 2–4 cells layer deep; and 4, a ring of inflammatory cells >4 cells layer deep. To determine the extent of mucus production, quantified goblet cell hyperplasia in the airway epithelium using a five-point grading system: 0, no goblet cells; 1, <25%; 2, 25–50%; 3, 50–75%; and 4, >75%. Scoring of inflammatory cells and goblet cells was performed in at least 5 different fields for each lung section and mean score was calculated from 4–5 mice per group.

### Statistical analysis

Data were analyzed using Graphpad Prism 5 statistics program. Statistical analysis was performed using one-way ANOVA with Tukey's Multiple Comparison Test with error bars representing the mean ± standard error of mean (SEM). Differences between means were considered significant when P < 0.05.

## Results

### CIK cells are T cells endowed with both of Th1 and NK cell properties

**Fig 2:**
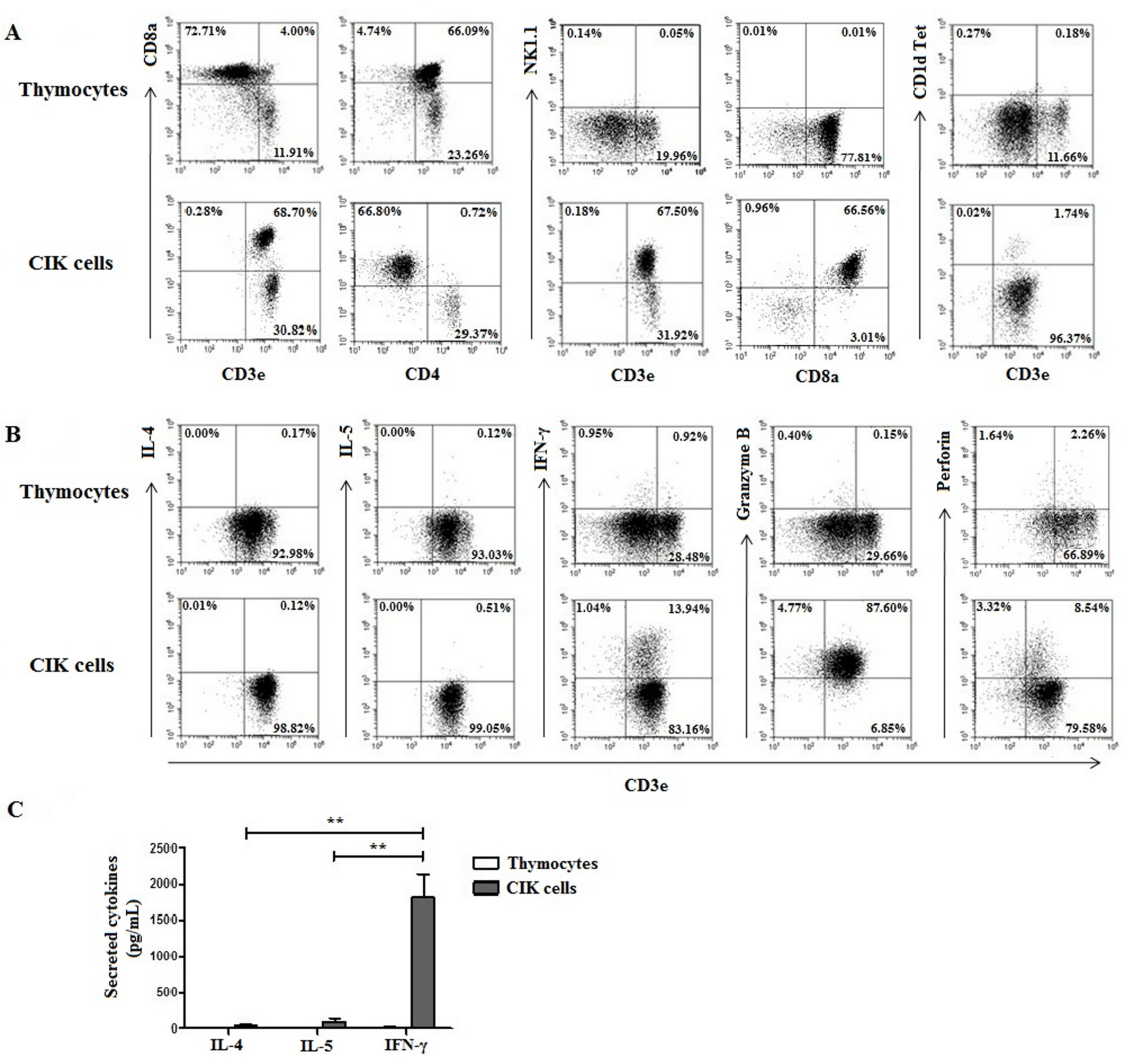
Phenotypic analysis of CIK cells. Thymocytes from C57BL/6 mice were cultured under the CIK culture condition as described in materials and methods. Flow cytometric analysis of thymocytes and CIK cells stained with antibodies to surface markers (CD3e, CD8a, CD4, NK1.1, α-GalCer–loaded CD1d tetramers) (A), cytokines (IL-4, IL-5, IFN-γ) and cytolytic granules (granzyme B, perforin) (B). Concentrations of cytokines (IL-4, IL-5, IFN-γ) in the supernatants collected from CIK cells or thymocytes suspended in the medium only for 48 hours. (C). Data shown are representative of 4 independent experiments. Data are means ± SEM (n = 4). (**, P<0.005).

As previously reported [2], thymocytes cultured under CIK culture condition resulted in the expansion of CD8^+^ NKT cells, and the majority of them were not CD1d-dependent NKT cells as confirmed by FACS staining with CD1d tetramer preloaded with α-GalCer (Fig 2A, S1 Fig). Additionally, not only these CIK cells are capable of producing markedly large amount of a Th1 cytokine (IFN-γ), not Th2 cytokines (IL-4, IL-5) (Fig 2B and 2C), but they also highly express cytolytic granules, granzyme B and perforin (Fig 2B). These results demonstrated that CIK cells possess both Th1 and NK characteristics.

### CIK cells could effectively suppress eosinophilic airway inflammation

**Fig 3:**
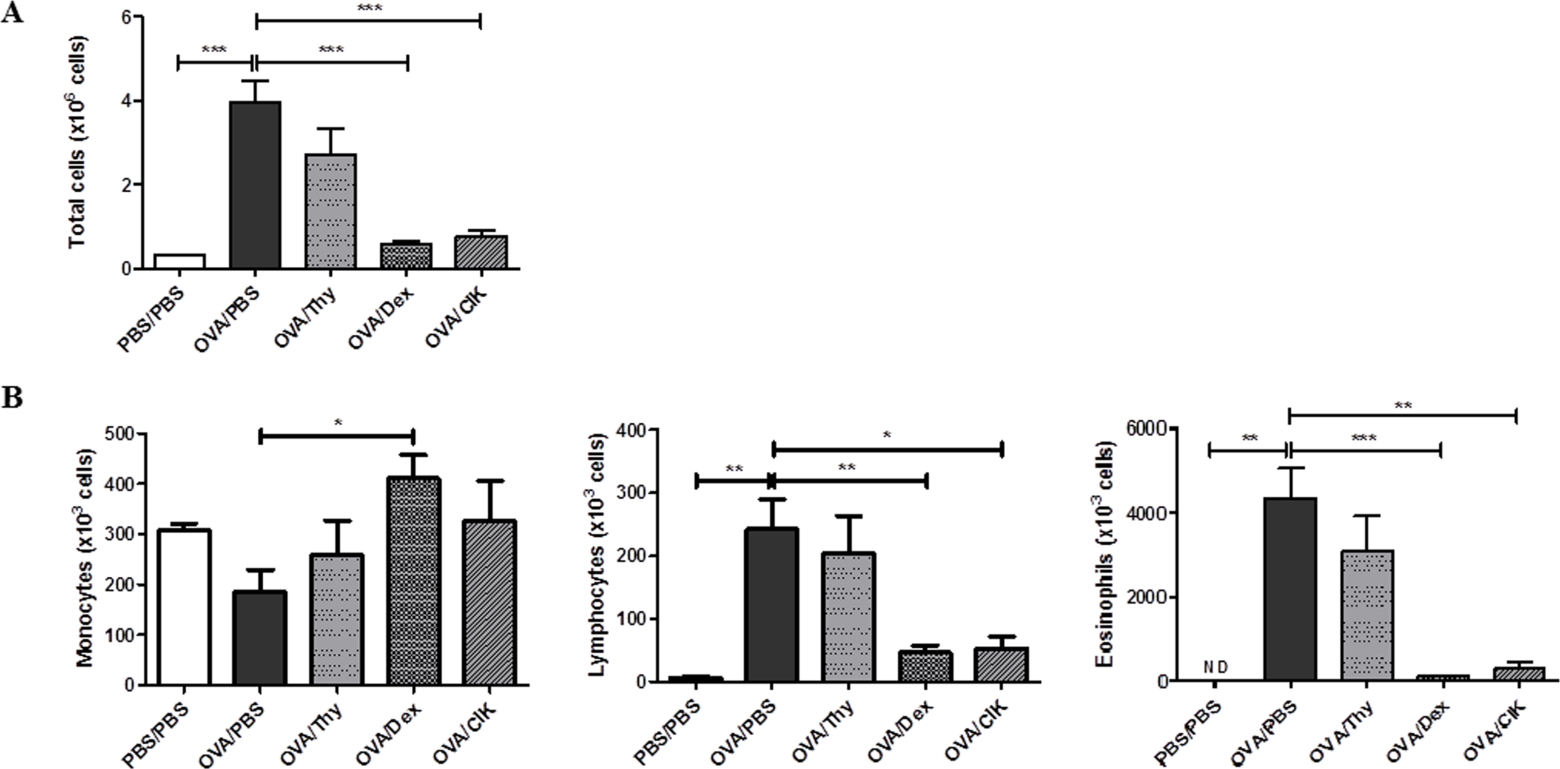
Cellularity of white blood cells in BAL. Total cell counts (A), numbers of monocytes, lymphocytes and eosinophils (B) were determined in BAL. Mice were sensitized and challenged with OVA and treated with PBS (OVA/PBS), thymocytes (OVA/Thy), dexamethasone (OVA/Dex) or CIK cells (OVA/CIK). Data are means ± SEM (n = 8–12) of 4 independent experiments. (ND, not detected; *, P<0.05; **, P<0.005 and ***, P<0.001).

To examine the therapeutic effect of CIK cells on the allergic airway inflammation using the mouse model of OVA-induced lung inflammation (Fig 1). The severity of allergic airway inflammation was determined by BAL total cell count and cellularity presenting with high proportion of eosinophils. OVA-sensitized mice treated with PBS (OVA/PBS), served as allergic control group, showed 15-fold raise in BAL total cell count compared to PBS-sensitized (PBS/PBS) normal control mice. Treatment with undifferentiated thymocytes (OVA/Thy) did not affect the severity of allergic airway inflammation, since there were no differences in BAL cell count and cellularity when compared to allergic control group (OVA/PBS). Treatment with CIK cells (OVA/CIK) dramatically reduced the BAL total cell count to the degree similar to dexamethasone treatment (OVA/Dex) (Fig 3A). As for BAL cellularity, the percentage of eosinophils in BAL decreased significantly and equally in both CIK and dexamethasone treatment groups, while the proportion of monocytes significantly increased (Fig 3B). This therapeutic effect of CIK cells could also be repeated in BALB/c mice (S2 Fig). Taken together, these results indicate that CIK cells can inhibit OVA-induced eosinophilic airway inflammation.

### Treatment with CIK cells suppressed Th2 cytokine productions in mice with allergic airway inflammation

**Fig 4:**
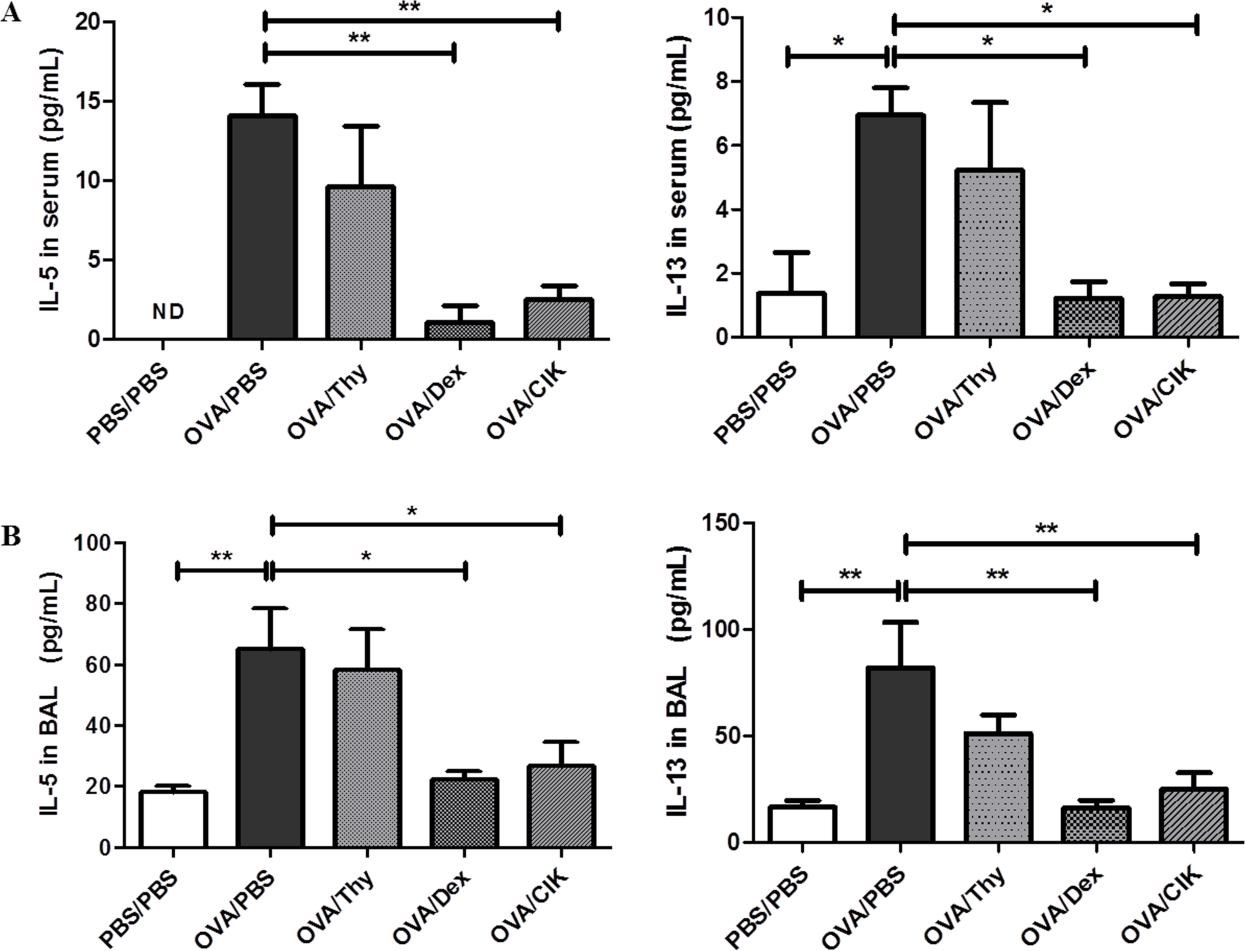
Th2 cytokine levels in serum and BAL. The amount of Th2 cytokines (IL-5, IL-13) in serum (A) and BAL (B) were measured in normal mice (PBS/PBS), OVA-sensitized mice treated with PBS (OVA/PBS), thymocytes (OVA/Thy), dexamethasone (OVA/Dex) or CIK cells (OVA/CIK). Data shown are means ± SEM (n = 4–8) of 4 independent experiments. (ND, not detected; *, P<0.05 and **, P<0.005).

Next, to further confirm the anti-allergic effect of CIK cells, the amount of allergic airway inflammation related Th2 cytokines, IL-5 and IL-13, in both serum and BAL were measured by ELISA (Fig 4A and 4B). A significant increase in the serum and BAL concentrations of IL-5 and IL-13 were observed in allergic control mice (OVA/PBS). Treatment of these allergic mice with CIK cells significantly decreased the productions of these Th2 cytokines. Moreover, the effect shown by CIK treatment was similar to dexamethasone treatment. Thus, we can conclude that CIK cells can attenuate the allergic airway inflammation through suppression of IL-5 and IL-13 productions.

### Treatment with CIK cells reduced inflammatory cell infiltration and goblet cell hyperplasia in the allergic lungs

**Fig 5:**
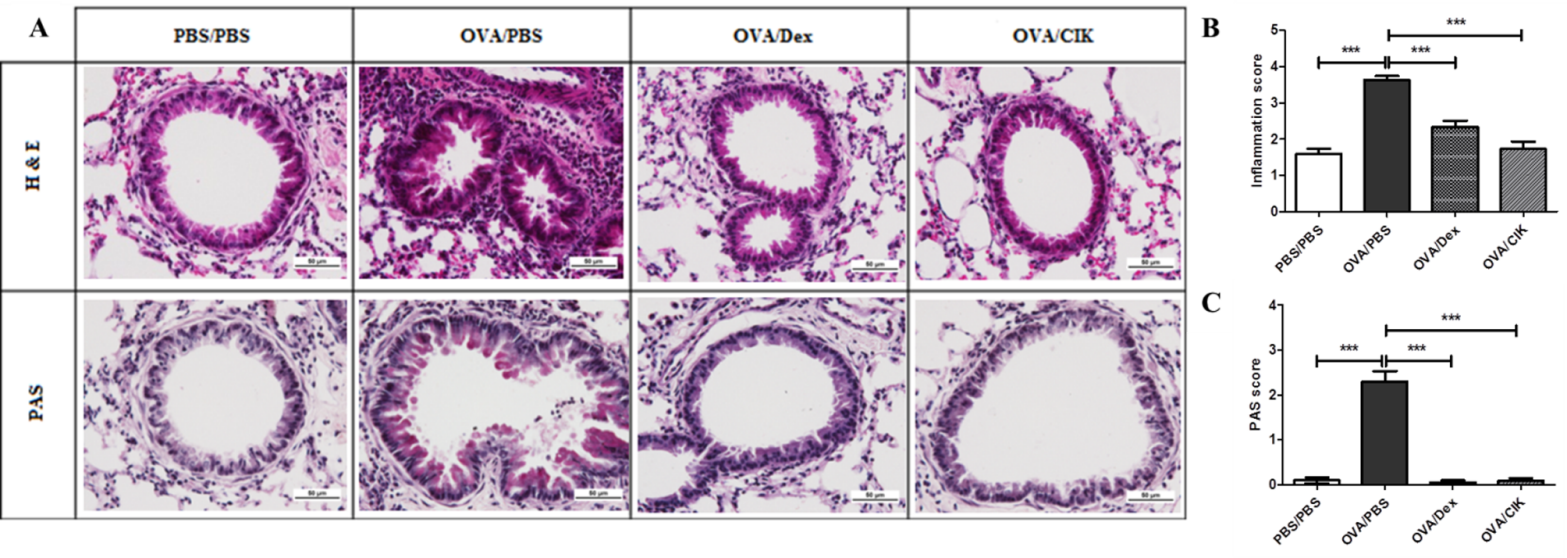
Photomicrographs of the lung section. Paraffin-embedded lung sections stained with H&E (upper) and PAS (lower) from normal mice (PBS/PBS), mice with allergic airway inflammation treated with PBS (OVA/PBS), dexamethasone (OVA/Dex) or CIK cells (OVA/CIK) (A). Bar graph represents semi quantitative scoring of inflammatory cells Infiltration (mean ± SEM) (B) and PAS positive cells (mean ± SEM) (C). Scores were obtained from 3 animals/group. (***, P<0.001).

The lung histology is another hallmark to determine severity of allergic airway inflammation. Accordingly, Lung sections of OVA/PBS mice showed increases in inflammatory cell infiltration and mucus producing goblet cells, while those from OVA/CIK and OVA/Dex mice similarly exhibit markedly reductions in both inflammatory cell infiltration and goblet cell hyperplasia (Fig 5A). The observations were confirmed by semi quantitative scoring of the inflammatory cell infiltration and PAS positive cells (Fig 5B and 5C). This lung histological evaluation further confirmed that CIK cell treatment could alleviate severity of allergic airway inflammation.

## Discussion

Allergic airway inflammation is a chronic inflammatory disease of the lungs characterized by recruitment of inflammatory cells into the lung and increased production of Th2 cytokines [12,13]. One possible therapeutic strategy to treat allergic airway inflammation is based upon Th2 counteractivity. Here, we hypothesized that CIK cells possessing both Th1 and NK cell properties could ameliorate allergic airway inflammation. CIK cells derived from thymocytes exhibited both Th1 and NK characteristics as evidenced by high productions of Th1 cytokines (IFN-γ) and cytolytic molecules (granzyme B and perforin), respectively. This study provides the first experimental evidence demonstrating that CIK cells alleviate OVA-induced allergic airway inflammation in mice. Mice with allergic airway inflammation treated with CIK cells exhibited significant reductions in important hallmarks of allergic airway inflammation; eosinophils in BAL, Th2 cytokines (IL-5, IL-13) production, inflammatory cell infiltration and mucus secreting cells in the airways.

IL-5 and IL-13 produced by CD4+ T cells in the airways have been shown to play pivotal roles in maturation and differentiation of eosinophils and recruitment of eosinophils into the lungs, subepithelial fibrosis and goblet cell hyperplasia [22–24], respectively. Here, we showed that, following CIK cells infusion, the elevated levels of both Th2 cytokines in the serum and BAL along with reduction in numbers of eosinophils in BAL in OVA-challenged mice. Accordingly, treatment with CIK cells could effectively diminish allergic airway inflammation; in particular, inflammatory cell infiltration and goblet cell hyperplasia in the airways to the degree similar to dexamethasone treatment through suppression of these Th2 cytokine productions. It is possible that high production of Th1 cytokine, IFN-γ [16] and cytotoxicity [19] by CIK cells contribute to their anti-allergic effects. Although CIK cells have long been proven to be safe and effective strategy to treat cancers in several clinical trials [5–10], little did we know about the cellular physiology of CIK cells. Interestingly, using CD1d tetramer preloaded with α-GalCer, we could confirm that major effector cells of CIK cells are CD8^+^ T cells expressing NK cell markers (CD8^+^ NKT-like cells), not CD1d-dependent NKT cells, as previously reported [2,25]. It is of importance to further explore the physiology of these CD8^+^ NKT-like cells. Collectively, our findings suggest that CIK cells can be applied to treat allergic airway inflammation.

Considering patients with allergic airway inflammation, the prevalence of severe asthma is estimated to vary from 4% to 10% of all the patients. These patients suffer from substantial morbidity because of poor control and several adverse effects due to prolonged treatment with high doses of corticosteroids [26]. We hope that our novel therapeutic approach using CIK cells to treat allergic airway inflammation will be beneficial especially for patients with severe asthma in the future.

## Conclusion

This study showed for the first time that CIK cells can be used to treat allergic airway inflammation based on the mouse model of OVA-induced airway inflammation. Treatment with CIK cells effectively suppressed eosinophilic airway inflammation to the degree similar to dexamethasone treatment. Further clinical studies are required to confirm this application in clinical practice. We hope that CIK cells can be an effective immunotherapy for allergic airway inflammation in the future.

## Supporting information

S1 Fig Flow cytometry gating. Flow cytometry gating of all fluorochrome-labeled antibodies to CD3e, CD4, CD8a, NK1.1, IL-4, IL-5, IFN-γ, granzyme B, perforin and R-PE conjugated CD1d tetramers preloaded with α-GalCer with the respective isotype control antibodies and CD1d tetramer negative control.
(TIF)

S2 Fig Cellularity of white blood cells in BAL using BALB/c mice. Total cell counts in BAL were determined (A). The percentages (B) and numbers (C) of monocytes, lymphocytes and eosinophils in BAL were determined. BALB/c mice were sensitized and challenged with OVA and treated with PBS (OVA/PBS) or CIK cells (OVA/CIK). Data are means ± SEM of 6–7 mice/group done in triplicate (*, P<0.05 and **, P<0.005).
(TIF)
