# Supplementary figures and images for: Cytokine-induced killer cells: A novel treatment for allergic airway inflammation
Source: PLoS One. 2017 Oct 26;12(10):e0186971. doi: 10.1371/journal.pone.0186971 (PMC5658108; doi:10.1371/journal.pone.0186971)

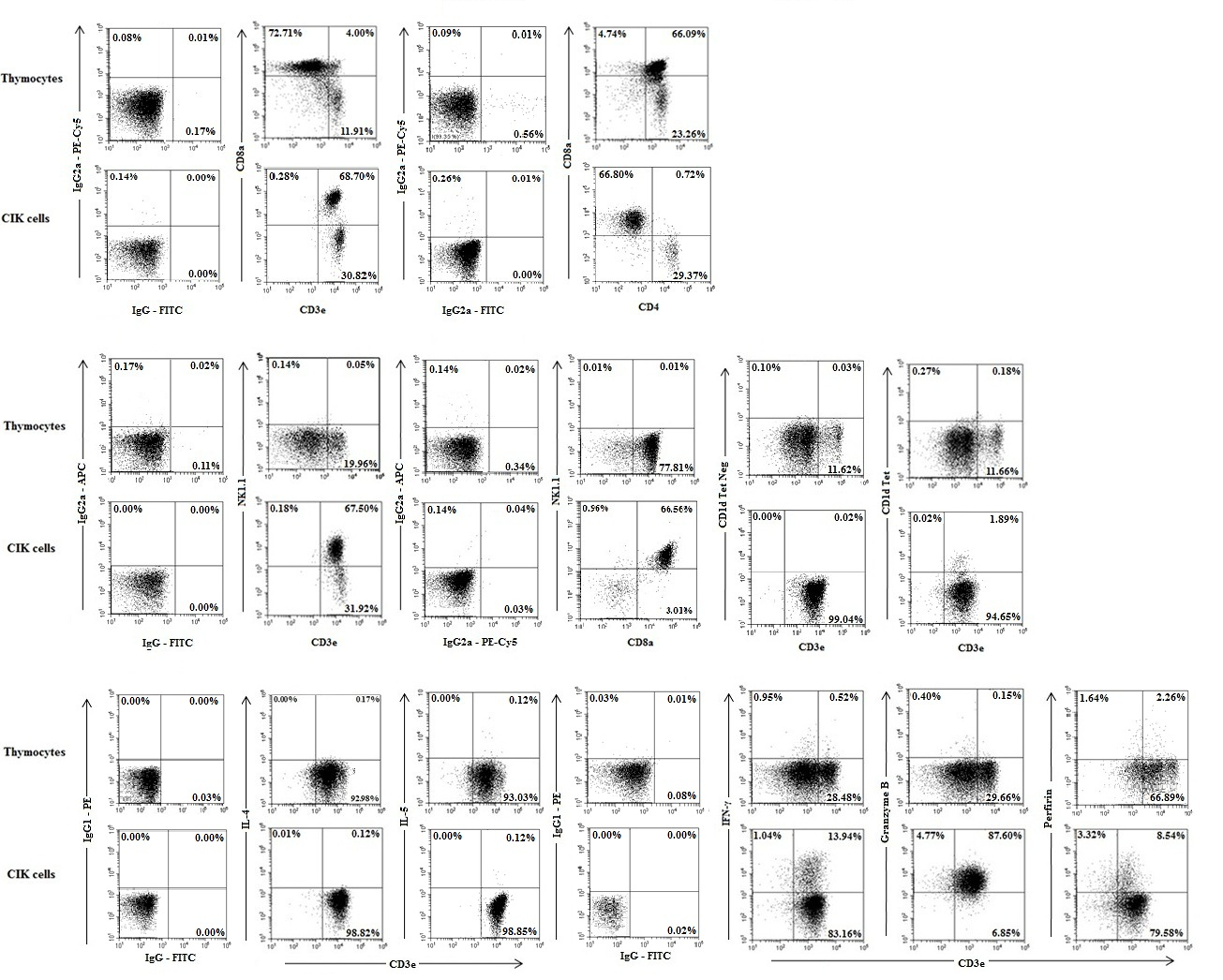

Supplement: S1 Fig — Flow cytometry gating of all fluorochrome-labeled antibodies to CD3e, CD4, CD8a, NK1.1, IL-4, IL-5, IFN-γ, granzyme B, perforin and R-PE conjugated CD1d tetramers preloaded with α-GalCer with the respective isotype control antibodies and CD1d tetramer negative control. (TIF) [file pone.0186971.s001.tif]

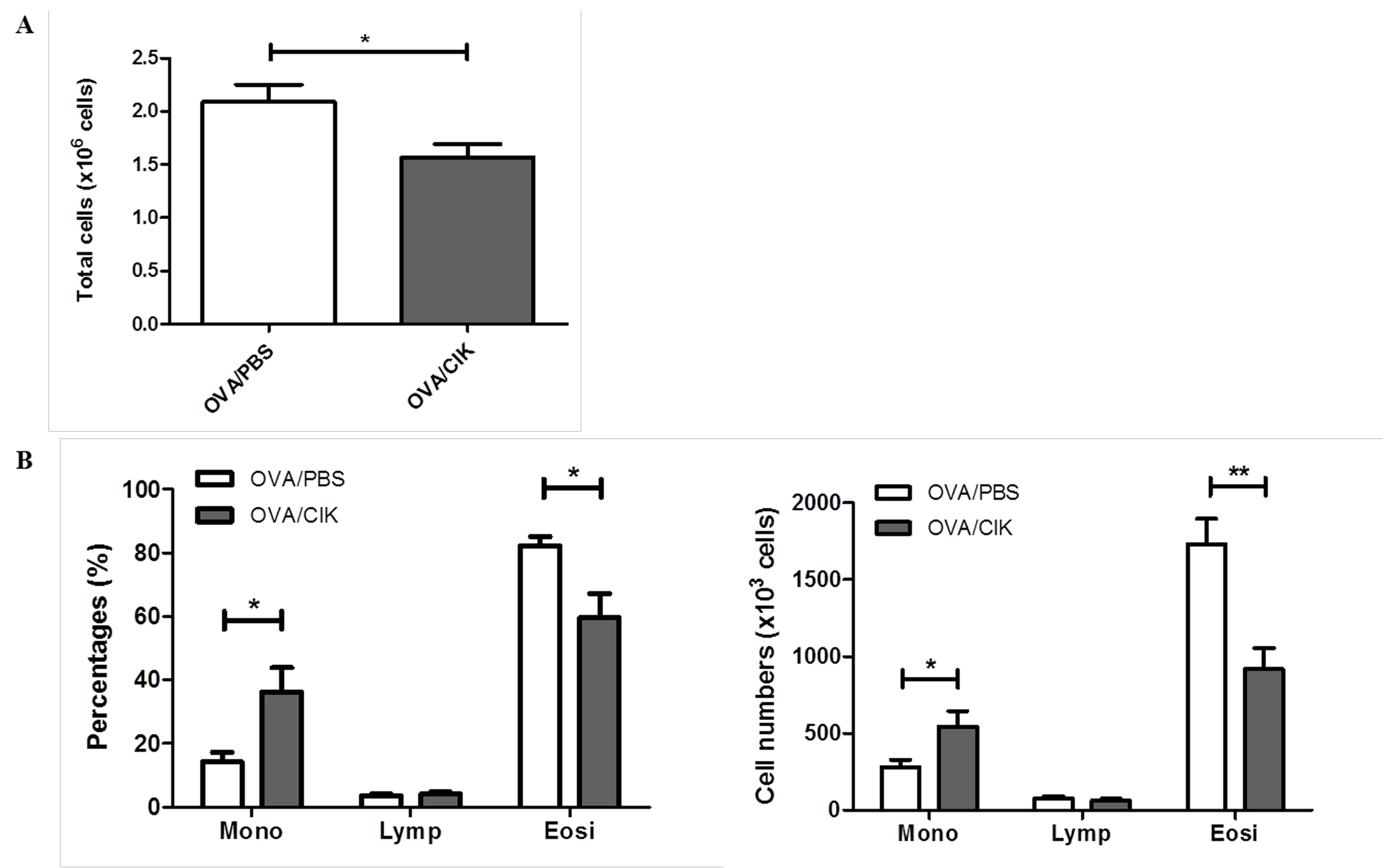

Supplement: S2 Fig — Total cell counts in BAL were determined (A). The percentages (B) and numbers (C) of monocytes, lymphocytes and eosinophils in BAL were determined. BALB/c mice were sensitized and challenged with OVA and treated with PBS (OVA/PBS) or CIK cells (OVA/CIK). Data are means ± SEM of 6–7 mice/group done in triplicate (*, P<0.05 and **, P<0.005). (TIF) [file pone.0186971.s002.tif]
